# Hypertensive Crisis in Acute Cerebrovascular Diseases Presenting at the Emergency Department: A Narrative Review

**DOI:** 10.3390/brainsci11010070

**Published:** 2021-01-07

**Authors:** Mariagiovanna Cantone, Giuseppe Lanza, Valentina Puglisi, Luisa Vinciguerra, Jaime Mandelli, Francesco Fisicaro, Manuela Pennisi, Rita Bella, Rosella Ciurleo, Alessia Bramanti

**Affiliations:** 1 Department of Neurology, Sant’Elia Hospital, ASP Caltanissetta, Via Luigi Russo, 6, 93100 Caltanissetta, Italy; m.cantone@asp.cl.it; 2 Department of Surgery and Medical-Surgical Specialties, University of Catania, Via Santa Sofia, 78, 95123 Catania, Italy; 3 Department of Neurology IC, Oasi Research Institute—IRCCS, Via Conte Ruggero, 73, 94018 Troina, Italy; 4 Department of Neurology and Stroke Unit, ASST Cremona, Viale Concordia, 1, 26100 Cremona, Italy; valentina.puglisi@asst-cremona.it (V.P.); luisa.vinciguerra@asst-cremona.it (L.V.); 5 Department of Neurosurgery, Sant’Elia Hospital, ASP Caltanissetta, Via Luigi Russo, 6, 93100 Caltanissetta, Italy; j.mandelli@asp.cl.it; 6 Department of Biomedical and Biotechnological Sciences, University of Catania, Via Santa Sofia, 89, 95123 Catania, Italy; drfrancescofisicaro@gmail.com (F.F.); manuela.pennisi@unict.it (M.P.); 7 Department of Medical and Surgical Sciences and Advanced Technologies, University of Catania, Via Santa Sofia, 78, 95123 Catania, Italy; rbella@unict.it; 8 IRCCS Centro Neurolesi Bonino-Pulejo, S.S. 113, Via Palermo C/da Casazza, 98123 Messina, Italy; rossella.ciurleo@irccsme.it (R.C.); alessia.bramanti@gmail.com (A.B.)

**Keywords:** ischemic stroke, hemorrhagic stroke, cerebrovascular events, arterial hypertension, hypertensive crisis, management, drugs

## Abstract

Hypertensive crisis, defined as an increase in systolic blood pressure >179 mmHg or diastolic blood pressure >109 mmHg, typically causes end-organ damage; the brain is an elective and early target, among others. The strong relationship between arterial hypertension and cerebrovascular diseases is supported by extensive evidence, with hypertension being the main modifiable risk factor for both ischemic and hemorrhagic stroke, especially when it is uncontrolled or rapidly increasing. However, despite the large amount of data on the preventive strategies and therapeutic measures that can be adopted, the management of high BP in patients with acute cerebrovascular diseases presenting at the emergency department is still an area of debate. Overall, the outcome of stroke patients with high blood pressure values basically depends on the occurrence of hypertensive emergency or hypertensive urgency, the treatment regimen adopted, the drug dosages and their timing, and certain stroke features. In this narrative review, we provide a timely update on the current treatment, debated issues, and future directions related to hypertensive crisis in patients referred to the emergency department because of an acute cerebrovascular event. This will also focus greater attention on the management of certain stroke-related, time-dependent interventions, such as intravenous thrombolysis and mechanic thrombectomy.

## 1. Introduction

### 1.1. Arterial Hypertension

Arterial hypertension is the main risk factor for cardio- and cerebrovascular diseases in Western countries [1,2,3,4,5,6]. It is estimated that the majority of patients presenting at the emergency department (ED) with hypertensive crisis, defined as an increase in systolic blood pressure (SBP) higher than 179 mmHg or diastolic blood pressure (DBP) higher than 109 mmHg [7], have had poor control of the arterial BP in the previous months. Epidemiological data also show that only 17.4% of patients with arterial hypertension achieve adequate BP control [8,9].

In recent years, progress has been made in understanding the pathophysiology of hypertension and its complications. In particular, solid evidence has demonstrated that BP control significantly reduces morbidity and premature mortality [10,11,12,13]. However, the control rates remain rather low and still far from being satisfactory worldwide. In neurological practice, a history of hypertension or uncontrolled BP commonly occurs at stroke onset, which is also associated with adverse clinical outcomes, early recurrent events, and high mortality [14].

Nowadays, several strategies, based both on healthy lifestyle and pharmacological treatment, are highly effective and well tolerated in the reduction of high BP values. Nevertheless, despite the amount of data on the impact of hypertension in stroke and the need for prevention, the management of BP during the acute phase of both ischemic and hemorrhagic stroke remains an area of debate. Moreover, the relationship between BP and cardiovascular events means the distinction between normal BP and hypertension in still somewhat based on arbitrary cut-off values. As shown in several clinical trials [15,16,17], hypertension is defined as the level of BP at which the benefits of treatment, achieved with lifestyle or pharmacological interventions, unequivocally overcome the risks of the treatment itself. This evidence provides the basis for the definition of hypertension and for the classification of BP severity [8].

Traditionally, hypertension has been defined as an SBP ≥140 mmHg or DBP ≥90 mmHg [8]. These values are supported by several randomized, controlled trials demonstrating the beneficial effects of treating patients with the above-mentioned values. However, the latest report of the American College of Cardiology and the American Heart Association (AHA) Task Force [18] has significantly modified these threshold values by defining hypertension as a BP higher 130/80 mmHg and by considering normal BP as values lower than 120/80 mmHg. Moreover, within the hypertension classes, the task force also identified two stages, i.e., stage 1, defined as SBP 130–139 or DBP 80–89 mmHg; and stage 2, defined as BP ≥140/90 mmHg. Finally, the quantification of the total cardiovascular risk, i.e., the probability of an individual developing a cardiovascular event during a defined time interval, is a relevant part of the risk stratification and subsequent therapeutic choice for patients with arterial hypertension [18].

### 1.2. Hypertensive Emergency and Hypertensive Urgency

The guidelines of the European Society of Cardiology (ESC) on the management of hypertension and subsequent revisions [8,19] have provided the most used recommendations for the diagnosis and treatment of hypertension, although they did not consider the difference between hypertensive emergency (HE) and hypertensive urgency (HU). In this framework, the classification of the Joint National Committee on Prevention, Detection, Evaluation, and Treatment of High Blood Pressure seems to be more accurate [7] by providing distinct definitions of HE and HU.

HE is characterized by a severe BP increase (generally > 180/120 mmHg) and is associated with acute organ damage, which includes acute cerebrovascular events, hypertensive encephalopathy, aortic dissection, myocardial infarction, unstable angina, acute congestive heart failure with pulmonary edema, hemolysis, increase in liver enzymes and low platelet count (HELLP syndrome), eclampsia, acute renal failure, and microangiopathic hemolytic anemia. Overall, hypertensive crises represent approximately 0.5% of all cases presenting at the ED [20]. A hypertensive crisis can develop in a previously normal subject or can complicate an underlying hypertension. Any medical, neurological, or psychiatric disorder causing a sudden rise of BP can result in an HE. Notably, a preexisting hypertension can reduce the probability of HE through some adaptive vascular modifications that seem to protect organs from constantly high BP values [21,22]. Conversely, in HU organ damage is not present or it is minimal, or is not clearly evident. Examples of HU, in which BP values are equal to or greater than grade 2 (SBP 160–179 mmHg or DBP 100–109 mmHg), are severe migraine, epistaxis, dyspnea, or severe anxiety [23,24]. Therefore, it is evident that a differentiation between HE and HU is crucial for management and optimal therapeutic approaches. Moreover, unlike HE, HU can be resolved spontaneously or within 24–48 h by using oral or intramuscular medications [25].

However, in the common scenario of BP > 180/120 mmHg at the ED, an acute hypertension may be classified as either HE or HU, since it is difficult to differentiate them based on BP values only, without clinical evidence of organ damage. The relatively paucity of data regarding this distinction is reflected in the fact that the 2014 Eighth Joint National Committee guidelines for the management of high BP in adults did not even mention HU and HE [26]. The term “urgency” has also led to overly aggressive management of many patients with severe but uncomplicated hypertension [7], to such an extent that the policy statement of the American College of Emergency Physicians in 2013 proposed the term “asymptomatic elevated BP” rather than HU [27]. Accordingly, even severe BP elevations without acute organ dysfunction should not be considered an HE, which conversely is characterized by impending or progressive organ damage in the context of high or very high BP (usually >180/120 mmHg) [28]. Additionally, the rate and magnitude of an increase in BP may be at least as important as the absolute level of BP in determining the severity of the organ injury [29].

To date, the pathophysiology of HE and HU is not completely understood, but it is likely that the increase in vascular resistance caused by angiotensin II, noradrenaline, or other endogenous compounds plays a crucial role. The renin–angiotensin–aldosterone system is involved in the development of severe hypertension, and some polymorphisms of the angiotensin-converting enzymes (ACE) or angiotensinogen encoding genes are associated with early onset hypertension [30,31]. Additionally, the levels of circulating catecholamines are associated with a hypertensive state, especially under stress conditions and in young patients. The induced state of sympathetic hyperactivation causes an increase in peripheral resistance and a decrease in arterial wall compliance [32]. During the initial increase in BP, the endothelium attempts to compensate by releasing vasodilatory substances such as nitric oxide. When hypertension is sustained, these mechanisms are overwhelmed and the result is an increase in BP, with subsequent endothelial damage. Thus, a vicious circle of hemodynamic and vascular damage is engaged, with a progressive increase in resistance and further endothelial damage, including the blood–brain barrier (BBB) [32].

Although the exact cellular processes leading to the loss of endothelial function in hypertensive crisis are only partially known, it is likely that these mechanisms involve proinflammatory responses, including the release of cytokines and chemotactic monocyte proteins, an increased concentration of intracellular calcium, a release of the vasoconstrictor agent endothelin-1, and the up-regulation of adhesion molecules in vascular cells [32]. Further, it should be noted that angiotensin II is able to induce the transcription of genes encoding for proinflammatory cytokines, thus contributing to the vascular damage [33]. Eventually, these molecular events will trigger excessive vascular permeability, inhibit local endothelial fibrinolytic activity, and activate the coagulation cascade. Platelet aggregation and degranulation on the damaged endothelium promote inflammatory, thrombotic, and vasoconstrictive phenomena, which self-maintain the pathological process [22]. Additionally, the brain parenchyma plays a relevant role in BP control through so-called cerebral autoregulation. In particular, the rostral ventrolateral medulla and the upper cervical spinal cord centers, modulated by the higher brain regions, are implicated in the control of BP [34].

The management of patients with HU or HE considerably varies in the context of organ damage or an acute cerebrovascular event. The organ damage secondary to increased BP can manifest in different ways and the treatment is based on a large number of drugs, which can be administered per os or via parenteral routes, usually intravenously (IV). The efficacy of the ACE inhibitors, angiotensin II receptor antagonist, calcium channel blockers, α-blockers, β-blockers, diuretics, nitrates, dopamine agonists, or their combinations is well known. The patient with HU can be safely discharged after observation in the ED and followed-up over time with periodical therapeutic changes if necessary. The management of HE is more complex since the patient faces both organ damage and significant alteration of the BP self-regulation system [22,35]. Management involves the administration of specific drugs with a short onset of action, short half-life, and which are easily titratable. Currently, there is no evidence showing the superiority of a drug class in terms of reduction of mortality and morbidity in the course of HE, with the pharmacological choice still being guided by the patient’s clinical presentation and drug availability [36].

To summarize, acute hypertension is categorized as HU or HE depending on the absence or presence of one or more target organ damage factors, respectively. Although scarce data exist regarding the evidence-based management, current European and USA guidelines recommend initiation, re-initiation, or intensification of oral antihypertensive therapy for HU, whereas controlled IV antihypertensive therapy is reserved for HE. The optimal medication, route of administration, and rapidity with which optimal BP needs to be achieved depend on the type of end-organ damage.

### 1.3. Acute Cerebrovascular Diseases and Arterial Hypertension

The typical acute cerebrovascular complication of arterial hypertension is a cerebral stroke, either ischemic or hemorrhagic. Stroke is the most common cause of disability, the second most common cause of dementia, and the third most common cause of death worldwide, representing an ongoing challenge for therapy and rehabilitation [37]. In Western countries, approximately 80–85% of all strokes are of ischemic origin, which develop when significantly reduced or interrupted cerebral blood flow (CBF) rapidly occurs in an area of the brain. According to the World Health Organization, stroke is an “acute neurovascular syndrome characterized by the sudden and rapid development of symptoms and signs referable to focal deficits of brain functions without any other apparent cause than the vascular one; the loss of brain function may be global (cerebral coma). The symptoms last more than 24 h or cause death” [38].

Ischemic strokes may be caused by several pathomechanisms, including large artery atherosclerotic disease resulting in stenosis or occlusion, small vessel penetrating artery disease, cardiogenic or artery-to-artery embolism, non-atherosclerotic vasculopathies, hypercoagulable disorders, or undetermined causes. A transient ischemic attack (TIA) is a focal neurological deficit of sudden onset caused by ischemia of the brain, retina, or cochlea lasting less than 24 h and followed by complete recovery, and is a strong prognostic factor of stroke. However, given that during diffusion-weighted scans involving magnetic resonance imaging (MRI) of the brain, a substantial percentage of patients clinically diagnosed as TIA exhibit a permanent ischemic lesion, the definition of TIA has moved from being time-based to tissue-based [39]. Arterial hypertension is a predisposing factor for both TIA and ischemic stroke by aggravating atherosclerosis and accelerating heart disease, increasing the relative risk of cerebral ischemia 3–4-fold. Hypertension also worsens the stroke outcomes, since patients with preexisting hypertension have smaller penumbra and larger infarction compared with those with normal BP [40].

Intracerebral hemorrhage (ICH) is the second most common subtype of stroke and accounts for approximately 15–20% of stroke cases [41], whereas subarachnoid hemorrhages do not exceed 5%. The incidence of ICH is substantially variable across countries and ethnicities and increases with advanced age [42]. The main cause of ICH is the rupture of small penetrating arteries secondary to hypertensive increase or other vascular abnormalities [43], causing arteries to show prominent degeneration of the tunica media and smooth muscle cells [44], whereas fibrinoid necrosis of the subendothelium with microaneurysms and focal dilatations may be seen in some patients. Lipohyalinosis, which is prominently related to long-standing hypertension, is most often found in non-lobar ICH [45]. Hypertension is the most important risk factor for spontaneous ICH, with a greater contribution to deep ICH than for lobar ICH [46].

Aside from cerebrovascular events, a common neurological complication of high BP is hypertensive encephalopathy (HTE). Typical symptoms are headache, mental confusion, visual disturbances, and sweating [47], thus meaning this condition can be promptly identified and managed at the ED. Evidence shows that HTE is a reversible condition that is usually resolved with adequate BP control [48]. Therefore, the average BP should be reduced by 20–25% within the first 4 h from the clinical onset and gradually reduced further in the next 24 h. Two different hypotheses have been proposed to explain its pathogenesis. One states that a vasospasm in response to a sudden increase in BP would result in the reduction of CBF, and eventually in intra-arterial thrombosis with secondary ischemia of the downstream territories [49,50,51]. An alternative and more convincing explanation states that HTE may result from excessive distension of the cerebral vessels, with subsequent fluid extravasation in the interstitial space and secondary edema and ischemia of the downstream territories [49,50,51]. The gold standard diagnostic method is MRI, which shows T2-weighted images of increased signal intensity [22], more often located in the occipital and parieto-occipital lobes, superior frontal regions, cerebellum, and basal ganglia [52].

A particular presentation of HTE is the so-called posterior reversible leukoencephalopathy syndrome (PRES), which typically involves the subcortical white matter of the occipital and parietal lobes [53]. This condition differs from classical HTE, since it can occur even in the absence of a BP increase [54]. The underlying mechanism consists of a loss of the self-regulative mechanisms of CBF and the disruption of the BBB, eventually resulting in fluid transudation from arterial vessels and cerebral edema [55]. In PRES with normal BP values, disruption of the BBB is probably due to the effects of specific harmful components on endothelial cells, such as sepsis or some drugs [55]. The differential diagnosis with PRES is essentially based on clinical examination and MRI findings [55,56,57].

### 1.4. Aim

In this narrative review, we provided a timely update on current treatment, debated issues, and future directions for hypertensive crisis in patients referred to the ED because of acute cerebrovascular events. This will also focus greater attention on the management of certain stroke-related, time-dependent interventions, such as IV thrombolysis and mechanic thrombectomy.

## 2. Data Source and Selection

A literature review was performed in relevant databases (i.e., MEDLINE (PubMed), Scopus, Web of Science, and Embase) to identify all studies published on hypertensive crisis and stroke. The following queries were performed from database inception to November 2020:

PubMed: ((hypertensive emergency) OR (hypertensive urgency)) AND ((ischemic stroke) OR (hemorrhagic stroke)). Filters: clinical study, clinical trial, controlled clinical trial, meta-analysis, observational study, randomized, controlled trial, systematic review, humans, English.

Scopus: TITLE-ABS-KEY (((hypertensive AND emergency) OR (hypertensive AND urgency)) AND ((ischemic AND stroke) OR (hemorrhagic AND stroke))) AND (LIMIT-TO (DOCTYPE, “ar”)) AND (LIMIT-TO (LANGUAGE, “English”)) AND (LIMIT-TO (SRCTYPE, “j”)).

Web of Science: TOPIC: (((hypertensive emergency) OR (hypertensive urgency)) AND ((ischemic stroke) OR (hemorrhagic stroke))) Refined by: LANGUAGES: (ENGLISH) AND DOCUMENT TYPES: (ARTICLE) AND LANGUAGES: (ENGLISH) Timespan: All years. Databases: WOS, BIOABS, BCI, DRCI, DIIDW, KJD, MEDLINE, RSCI, SCIELO, ZOOREC. Search language = Auto.

Embase: (“hypertensive emergency”/exp OR “hypertensive emergency” OR (hypertensive AND (:emergency”/exp OR emergency)) OR “hypertensive urgency”/exp OR “hypertensive urgency” OR (hypertensive AND (“urgency”/exp OR urgency))) AND “‘ischemic stroke”/exp OR “ischemic stroke” OR (ischemic AND (“stroke”/exp OR stroke)) OR “hemorrhagic stroke”/exp OR “hemorrhagic stroke” OR (hemorrhagic AND (“stroke”/exp OR stroke))) AND [article]/lim AND [English]/lim AND [humans]/lim.

**Figure 1:**
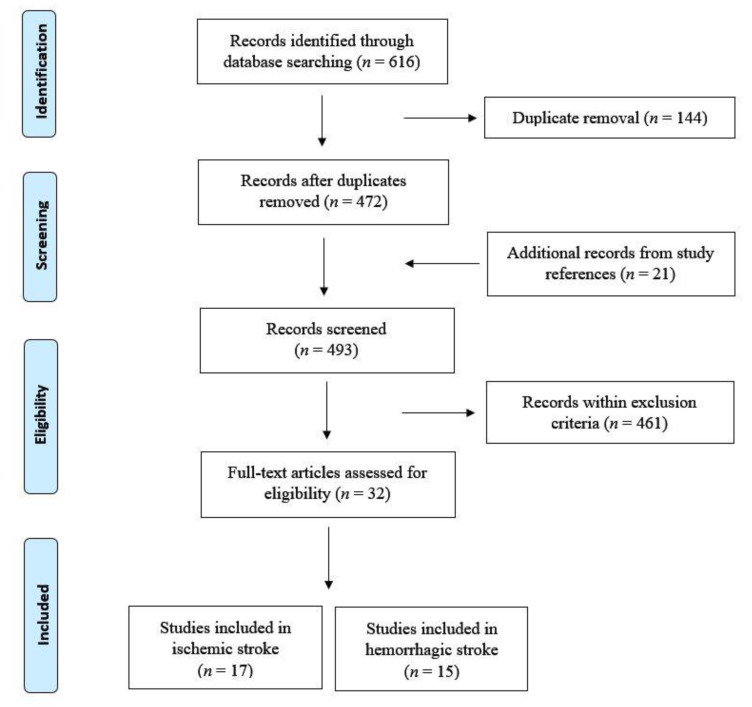
Flow diagram showing the search strategy, the number of records identified, and the numbers of included and excluded studies [58].

From an initial search using the above-mentioned keywords, a total of 616 articles were originally retrieved. After removal of duplicates (*n* = 144), the results were further screened to exclude all studies conducted on animals or cell cultures, preliminary reports and publications different from research studies (e.g., reviews, letters, editorials, and commentaries), studies on pregnant women or children, non-English papers, and any other studies that did not fit with the aim of this review (*n* = 461). Articles listed in the reference were also reviewed in search of more data (*n* = 21). At the end of this process, 17 studies on ischemic stroke (Table 1) and 15 on hemorrhagic stroke (Table 2) were selected and included in the analysis. A flow diagram of the included/excluded studies is shown in Figure 1.

For each study, the following data were extracted: (a) study design; (b) patient characteristics, such as sample size, age, sex, diagnostic criteria used, and stroke type; (c) drug or drugs used; (d) any thrombolytic or thrombectomy therapy performed.

## 3. Results

### 3.1. Ischemic Stroke

Based on the evidence reviewed here from patients receiving IV thrombolysis, BP in ischemic stroke should be lowered and maintained at lower than 180/105 mmHg for at least the first 24 h after thrombolysis [66,73]. Some observational data, such as the post hoc analysis of the International Stroke Trial, indicate a strong association between BP control and clinical outcome, with increased mortality and disability in cases of either high- or low-level ranges of BP [14]. In the National Institute of Neurological Disorders and Stroke-recombinant tissue plasminogen activator (NINDS-rtPA) stroke trial, the frequency of hypertension and the use of antihypertensive therapy were similar between the rtPA and placebo groups. In particular, in the placebo group, antihypertensive therapy was not associated with less favorable outcomes at three months, whereas post-randomization antihypertensive therapy was associated with less favorable outcomes for the rtPA patients who were hypertensive. However, because of the non-randomized use of antihypertensive therapy and the many post hoc comparisons leading to type 1 errors, the significance of this observation remained unclear [60].

More controversial seems to be the benefit of a rapid BP reduction in patients with acute ischemic stroke who do not receive IV thrombolysis. As a general rule, both high and low SBP and DBP, as well as a relevant drop in BP, are associated with poor prognosis in patients with ischemic stroke in terms of neurological deterioration, infarct volume, neurological outcome, and mortality at three months [65]. Although several studies have addressed the BP reduction after an acute stroke [59,61,67,91], they did not have enough power to detect significant differences in mortality and disability. In the Study Treatment of Acute Hypertension (STAT) registry, median BP values at baseline were 183/95 mmHg, without any differences between survivors and non-survivors. However, patients who died had lower minimal BP values and mortality was also associated with an increased frequency of neurologic deterioration [92].

The Controlling Hypertension and Hypotension Immediately Post-Stroke (CHHIPS) study [67], which included both ischemic and hemorrhagic strokes, was designed to test the effects of changes of SBP up to 36 h from the stroke onset to mortality. The arm of the trial in which SBP was reduced showed that an early lowering of BP was the most effective approach to improving patients’ outcome. However, since the study included one patient only in the arm that increased BP, no conclusion can be drawn on the effects of this intervention. CHHIPS [67] was also the only study that specifically compared decreasing and increasing BP to find an ideal SBP target in the hyperacute phase (i.e., the first 24 h) of ischemic stroke. However, the study was ended early due to recruitment difficulties. Another study, the China Antihypertensive Trial in Acute Ischemic Stroke (CATIS) trial [70], showed no benefit of SBP reduction within the first 48 h from ischemic stroke.

A recent meta-analysis [93] that included 26 studies and more than 17,000 participants concluded in favor of the reduction of BP during the acute phase (48 h) of stroke to improve functional outcome. However, although the early treatment (within 6 h) appeared effective in reducing functional dependence, it did not significantly affect mortality.

The Early Manipulation of Arterial Blood Pressure in Acute ischemic Stroke (MAPAS) study aimed to determine the effectiveness of early management of SBP in the first 24 h after an ischemic stroke that did not involve thrombolysis. The key assumption was that clinical outcomes varied across the range of variation in SBP in patients with ischemic stroke [75]. In detail, 218 patients were enrolled within 12 h from the symptom onset to maintain SBP during the following 24 h within three intervals (i.e., group 1: 140–160 mmHg; group 2: 161–180 mmHg; group 3: 181–200 mmHg). Drugs and vasoactive fluids were used to achieve these goals. The best outcome was defined as a modified Rankin scale score of 0–2 at 90 days. The median SBP values for the three groups at 24 h were 153 mm Hg, 163 mm Hg, and 178 mm Hg, respectively (*p* < 0.0001), while clinical outcomes did not differ among the groups (51% vs. 52% vs. 39%, respectively, *p* = 0.27). Symptomatic ICH occurred more frequently with the higher SBP interval (1.0% vs. 2.7% vs. 9.1%, respectively, *p* = 0.048), with similar mortality rates. No patient had neurological deterioration related to the BP reduction during the first 24 h [75]. During logistic regression analysis, the chances of a good clinical outcome were higher in group 2, including after adjusting for confounding factors. Regardless of the assigned group, the probability of a successful outcome was 47% in patients who underwent an increase of BP, 42% in those who had a decrease of BP, and 62% in those who were not manipulated (*p* = 0.1). Adverse effects were limited to groups 2 (4.0%) and 3 (7.6%) and were associated with the use of norepinephrine. The authors concluded that outcomes at 90 days did not significantly differ among the three ranges of BP values, and after logistic regression analysis, the odds of having a good result were higher in group 2 (i.e., SBP 161–180 mmHg) [75].

Hillis et al. [64] randomized patients with acute ischemic stroke with a large diffusion–perfusion mismatch to evaluate the effects of BP elevation in 9 patients (treated) compared with 6 (untreated) patients, and found better results among treated patients. Another study [63] on BP increase in 13 patients with ischemic stroke, regardless of the stroke subtype, found that 7 patients improved their neurological examination after BP manipulation. The Scandinavian Candesartan Acute Stroke Trial (SCAST) sub-analysis [72] showed that progressive clinical deterioration occurred more frequently in patients with ischemic stroke and stenosis of the internal carotid artery treated with antihypertensive drug, with an increased risk that paralleled the severity of stenosis (*p* = 0.04).

Regarding mortality and disability, a meta-analysis suggested that BP lowering immediately after an acute ischemic stroke had a neutral effect on prevention [94,95]. In patients with markedly elevated SBP or DBP (i.e., ≥220 or ≥120 mmHg, respectively), the clinical judgment should define whether the use of drugs, with a reasonable goal to lower BP by 15% and close monitoring, should be adopted during the first 24 h after the stroke onset [69,95,96,97]. Patients with acute ischemic stroke and BP lower than this value within the first 72 h did not seem to benefit from the administration or re-administration of antihypertensive drugs [71,95,97].

In clinically stable patients with hypertension (≥140/90 mmHg) for more than three days after an acute ischemic stroke, the antihypertensive drugs should be started or re-introduced [68]. However, although the autoregulation is lost in the territory affected by a cerebrovascular event [98], a positron emission tomography study (which measured the CBF before and after the reduction of BP) showed no significant differences in perfusion within the ischemic area, the peri-infarct region, and other non-affected regions in the ipsilateral hemisphere [99].

In this complex framework, in line with the European and American guidelines for hypertension management of ischemic stroke [29,100], the Italian guidelines for the emergency treatment of hypertension in patients with acute ischemic stroke (International Stroke Organization-Stroke Prevention and Educational Awareness Diffusion 2016, VIII edition) provide the following recommendations [101]:

(i) An automatic sphygmomanometer should be used instead of a manual device;
(ii) If DBP is >140 mmHg in two measurements within 5 min, a continuous IV infusion of antihypertensive agents such as nitroglycerin or sodium nitroprusside (0.5–1.0 mg/kg/min) should be started. Patients at risk for cerebral edema should be constantly monitored given the possibility of increase of the intracranial pressure. Such patients are not candidates for thrombolytic treatment;
(iii) If SBP is >220 mmHg, DBP is 121–140 mm Hg, or the mean BP is >130 mmHg in two measurements within 20 min, an antihypertensive drug that is easily titratable such as labetalol (10 mg IV in 1–2 min) should be administered. This dose can be repeated or doubled every 10–20 min up to a cumulative dosage of 300 mg. After this initial approach, labetalol can be administrated every 6–8 h if necessary. Labetalol is not recommended in patients with asthma, heart failure, or severe cardiac arrythmia. In these cases, urapidil (10–50 mg IV or infusion of 0.15–0.5 mg/min) should be considered. Patients requiring more than two doses of labetalol or any other antihypertensive drug to reduce SBP < 185 mmHg or DBP < 110 mmHg are generally not candidates for thrombolytic therapy;
(iv) When SBP is 185–220 mmHg or DBP is 105–120 mmHg, emergency therapy should be postponed if left ventricular failure, aortic dissection, or acute myocardial infarction coexist. Patients who are candidates for thrombolytic therapy who have persistently elevated SBP (>185 mmHg) or DBP (>110 mmHg) can be treated with small doses of IV antihypertensive drugs to maintain BP values below these limits. However, the administration of more than two doses of antihypertensive drugs to keep BP under control is currently considered as a relative contraindication to thrombolytic therapy [102];
(v) The use of calcium channel blockers by sublingual administration is not indicated due to the rapid and often unpredictable drop of BP that can be caused by this class of drugs;
(vi) Pharmacological BP correction in the acute stroke phase should be associated with careful monitoring of the neurological status in order to promptly detect any clinical deterioration;
(vii) In patients with acute ischemic stroke and SBP < 185 mmHg or DBP < 105 mmHg, antihypertensive therapy is not usually indicated [102];
(viii) Although there are no data to define a stable threshold, arterial hypotension in patients with acute stroke should be treated in cases of dehydration or significantly lower BP values for that patient. Treatment options include IV administration of fluids, treatment of an underlying congestive heart failure and bradycardia, and use of vasopressor agents such as dopamine [96,103].

### 3.2. Hemorrhagic Stroke

An acute hypertensive response is observed in up to 75% of patients with ICH [86]. The management of increased BP after the onset of ICH is still debated, and to date BP is lowered only when SBP exceeds 180 mmHg, targeting BP values of 160/90 mmHg [104]. It is known that increased BP may represent a constant stimulus for intracerebral bleeding, being associated with larger hematoma, neurological deterioration, and poor outcome [83,84,105]. Even early and intensive BP lowering in the acute phase of ICH was found to be effective and safe [76,77,80,81,82], suggesting that aggressive reduction might reduce the risk of neurological deterioration in the first 24 h of admission [78] and hematoma growth over 72 h, without appreciable effects on perihematomal edema [79]. Additionally, early initiation of oral antihypertensives (i.e., within 24 h versus after 24 h of ED arrival) reduced intensive care unit stays and hospital costs for patients with hypertensive ICH [90].

However, a subgroup analysis of the Efficacy of Nitric Oxide in Stroke (ENOS) trial showed that the continuation of antihypertensive drugs during the first week from clinical onset did not reduce death or major disability compared with stopping treatment temporarily [85]. Even the elevation of BP has been considered as a protective response, in order to maintain cerebral perfusion and prevent secondary cerebral ischemia. The multicenter prospective Antihypertensive Treatment of Acute Cerebral Hemorrhage (ATACH) phase I trial on 60 patients, which evaluated SBP reduction in three different target groups (i.e., first cohort: SBP 170–200 mmHg; second cohort: SBP 140–170 mmHg; third cohort: SBP 110–140 mmHg) showed the highest treatment failure, neurological deterioration, and a three-month mortality in the third cohort, although the study did not have sufficient power to demonstrate differences in three-month mortality or rates of clinical outcomes [80].

The multicenter, randomized, controlled phase III study, named the Second Intensive Blood Pressure Reduction in Acute Cerebral Haemorrhage Trial (INTERACT-2) trial, which was performed on 2839 ICH patients within 6 h of symptom onset, evaluated the safety and efficacy of aggressive BP lowering to values <140/90 mmHg within one hour compared to standard treatment (SBP < 180 mmHg) [81]. Overall, an absolute benefit of 3.6% in terms of the rate of severe disability or death with no effect on hematoma expansion was seen in the first group [81]. A subsequent meta-analysis [106] suggested possible functional recovery at three months after rapid IV reduction of SBP to less than 180 mmHg in these patients.

It is also worth mentioning that a subsequent meta-analysis evaluating the safety and efficacy of intensive BP reduction in patients with acute ICH showed no significant improvement in functional outcome at three months or in the growth of hematoma at 24 h compared to standard treatment [106]. In this scenario, the ATACH-2 trial [86] was a multicenter, randomized, controlled trial on 1000 patients undergoing intensive treatment (SBP < 140 mmHg) compared to standard treatment (SBP < 180 mmHg) within the first 4.5 h from ICH. Overall, there was no difference between the two groups, either in terms of reduction of hematoma, mortality, or disability at three months or in terms of secondary results, except for serious adverse events at three months in the intensive treatment group, including abdominal discomfort, limb and oral abscess, acute pulmonary edema, acute coronary syndrome, atrial fibrillation, agitation or anxiety, altered state of consciousness, brain edema, and anemia. Furthermore, the study showed a higher incidence of renal adverse events in the intensive treatment group compared to the standard treatment [86].

Another relevant issue is related to the neurosurgical treatment of patients with ICH. As the most common sites of spontaneous ICH are the deep brain structures, a large layer of brain tissue must be crossed during surgery, which may cause iatrogenic damage to the healthy brain. Post-surgical complications (e.g., hemorrhages and infections) are not uncommon in this scenario and often carry high rates of morbidity and mortality. One of the most important parameters to check during brain surgery is BP. During surgery, high BP causes diffuse bleeding, especially from capillaries, which is difficult to control and are often time-consuming for surgeons. In addition to bipolar forceps, there are many other hemostatic agents available, such as oxidized cellulose, gelatin foam, fibrillar collagen, fibrin sealants, antifibrinolytic [107], and the new intravenous mesenchymal stem cell therapy [108], although the control of high BP remains the best hemostatic approach. The consequences of non-optimal hemostasis are growth of the hematoma and ischemia from bipolar coagulation, eventually resulting in damage to healthy cerebral tissue and occurrence of additional neurological deficits.

In neurosurgical settings, it is also mandatory to check the international normalized ratio (INR) due to the frequent occurrence of hematoma growth. Although use of oral anticoagulants is increasing, there is a substantial lack of data on how to treat oral anticoagulant-associated ICH. Kuramatsu et al. [109] performed a retrospective cohort study from 19 German tertiary care centers involving 1176 individuals. Overall, they found that in patients with oral anticoagulants-related ICH, reversal of INR < 1.3 within 4 h and SBP < 160 mmHg were associated with lower rates of hematoma enlargement, whereas resumption of anticoagulant therapy was associated with lower risk of ischemic events without increased bleeding [109].

A treatment threshold of SBP 180 mmHg with a target goal of reduction to 130–150 mmHg within 6 h of symptom onset is also supported by the evidence recently provided by Qureshi et al. [110]. Based on these findings, the AHA, American Stroke Association (ASA) [105], and the Italian guidelines [101] recommended rapidly reaching (possibly within an hour) and maintaining (for at least 24 h, preferably for the first 7 days) an SBP < 140 mmHg in the acute phase of ICH. The drugs are the same as those used for the treatment of hypertension in ischemic stroke, whereas the choice and route of administration basically depend on the BP values and availability of the antihypertensive drugs.

Lastly, a recent systematic review of all randomized, controlled trials [111] evaluated the different effects of standard or intensive treatment of BP in patients with ICH. The primary outcomes were mortality at three months, disability based on a modified Rankin scale score, and the combination of death and disability. The secondary outcomes were deterioration of the neurologic state within 24 h, a significant ICH growth within 24–72 h, and non-fatal serious adverse events at three months. Six studies were included, with a total of 4385 patients (average age 62 years, 62.3% men). Globally, there were no differences between groups in terms of either primary or secondary outcomes, thus suggesting that the intensive control of BP in the acute phase of ICH was not beneficial and should not be recommended. Taking these considerations together, it is evident that optimal BP values in the acute phase of ICH have not been clarified yet [87,112,113] and that the conventional target of SBP < 140 mmHg, which is currently suggested by the international guidelines [101,105], should be revised [114].

## 4. Discussion

### 4.1. General Considerations

Hypertensive crisis typically causes end-organ damage; the brain is an early elective target, among others. The prognoses of patients with acute cerebrovascular diseases presenting at the ED with high BP differs mainly depend on HE or HU, the treatment regimen adopted, the drug dosages and their timing, as well as on some stroke features. The common goals of BP reduction in patients with acute cerebrovascular diseases include decreases of cerebral edema and of the risk of progression towards a hemorrhagic stroke, the prevention of further bleeding or hematoma growth, and reduction of the risk of recurrent strokes or other complications [115].

An acute increase of BP can be frequently observed within the first 24 h after an ischemic or hemorrhagic stroke [116], and 20% of subjects presenting with an acute hypertensive response associated with stroke do not have a previous history of arterial hypertension [117]. Overall, the increase in BP can be secondary to the stress caused by several factors, including the cerebrovascular event itself, a pre-existing hypertension, pain, urinary retention, or it can be a physiological response to hypoxia or increased intracranial pressure [98,116,118,119]. After the event, in the majority of patients, a decline in BP without any specific medical treatment can occur. Indeed, BP often falls spontaneously when the patient is moved to a quiet room, the bladder is emptied, pain is controlled, and the patient is allowed to rest.

The pathophysiology behind BP elevation following stroke remains poorly understood and different mechanisms have been proposed [120]. The self-limiting nature of this response suggests additional contributing factors other than merely inadequately treated hypertension [121,122]. In ischemic stroke, some studies have attributed the hypertensive response to ischemic injuries of different cerebral regions that are responsible for autonomic BP control [116]. Owing to the widespread locations of these areas, most strokes are thought to affect these regions to a varied extent. Other studies have suggested that the increase in BP may represent a beneficial response by maintaining perfusion through collateral branches in the presence of major vessel occlusion [64,123]. This hypothesis was supported by a study showing a steeper fall in BP in patients where recanalization was achieved as compared to those where major occlusions persisted [124]. Additionally, systemic factors, such as psychological stress [125], may be important contributors to the frequently elevated BP present on admission in patients with ischemic stroke [126]. The majority of stroke patients, particularly those with ICH, have chronic hypertension and elevation at the time of admission, which is merely a reflection of untreated hypertension [127], although it might also represent a reaction of the brainstem in order to maintain cerebral perfusion [44,128]. Stress responses leading to abnormal sympathetic activity, altered parasympathetic activity, and increased levels of circulating catecholamines [129] and brain natriuretic peptides may contribute to hypertension and to maintaining a hypertensive state [130].

Large BP variability also plays a major role in determining the outcome of stroke patients. A previous systematic review and meta-analysis [131] showed that long-term variability in BP was associated with cardiovascular and mortality outcomes, including stroke, over and above the effects of mean BP. Mid-term and short-term variability showed similar associations, although data are still limited [131]. It is known, indeed, that BP usually fluctuates within a few hours or over even more prolonged periods according to strict interactions between several components, such as environmental factors and cardiovascular regulatory mechanisms. In particular, BP variability increases with aging and with the occurrence of cardiovascular risk factors (e.g., diabetes, smoking habit, atrial fibrillation, and previous TIA or stroke) [132]. In patients with TIA or ischemic stroke, BP variability is associated with an increased risk of vascular events, including stroke, coronary artery disease, and heart failure [133,134]. In ICH patients, high in-hospital BP variability is associated with severe disability or death, especially elderly patients, female patients, and patients with high admission SBP [135]. In these cases, antihypertensive drugs should target a lowering of both mean BP and BP variability.

Lastly, hypertension is the most important modifiable risk factor for TIA, and therefore drugs lowering BP might play important roles in both primary and secondary stroke prevention. However, the relationship between BP and TIA is still not clear, especially in advancing age; conversely, increased SBP in patients younger than 80 with suspected TIA seems to be a useful clinical indicator upon initial presentation that could help in clinical diagnosis [136]. However, BP remains poorly controlled overall in a large proportion of patients after TIA, with under-treatment and poor adherence to treatment being relevant contributing factors [137]. To investigate whether BP-lowering drugs started at least 48 h after the index event were effective in the prevention of recurrent stroke, major vascular events, and dementia in people with stroke or TIA, a recent Cochrane systematic review of 11 randomized, controlled trials was conducted [138]. The secondary objectives were to identify subgroups of people in which drugs are effective and to investigate the optimum SBP target after stroke or TIA for preventing recurrent stroke, major vascular events, and dementia. The study included a total of 38,742 participants—eight studies compared BP-lowering drugs versus placebo or no treatment, whereas three studies compared different SBP targets. Although the risk of bias varied greatly between the studies, the results supported the use of BP-lowering drugs in patients with TIA or stroke for reducing the risk of stroke or recurrent stroke, respectively. The current evidence is primarily derived from trials studying ACE inhibitors or diuretics, although no definite conclusions can be drawn regarding an optimal SBP target after stroke or TIA [138].

### 4.2. Debated Issues

Although clinical trials have generally shown neutral effects of early BP decreases on clinical outcomes after acute ischemic stroke, the effects of early antihypertensive therapy for patients with ischemic stroke with or without prior hypertension are still unclear. Zhang et al. [139] recently performed a secondary analysis of the CATIS randomized trial among patients with acute ischemic stroke. In these subjects, early antihypertensive treatment was not associated with different death or major disability outcomes related to history of hypertension, although early antihypertension therapy decreased the rate of recurrent stroke in those with a history of hypertension [139].

Similarly, to date there is no good evidence of acute BP management in the pre-hospital setting of acute stroke [68,69,70], although the ultra-acute treatment of BP within the first few hours of symptoms in the pre-hospital setting would be of vital importance. Non-oral routes of administration, such as transdermal, sublingual, and IV, would be preferable in such cases, given the need for a swallowing assessment to rule out dysphagia [140]. Of these, transdermal glyceryl trinitrate [141], sublingual lisinopril [142], and IV magnesium [143] have been assessed in the pre-hospital environment and found to be safe. While transdermal preparations can be easily applied and removed according to clinical need, IV administration of BP-lowering agents requires intensive monitoring. Some on-going trials, e.g., the Rapid Intervention with Glyceryl Trinitrate in Hypertensive Stroke Trial (RIGHT-2) [144] and other planned investigations [145] of transdermal glyceryl trinitrate in ultra-acute stroke care, are assessing the safety and efficacy. In spontaneous ICH, several studies also showed that elevated pre-hospital SBP correlated with SBP on admission [89] and with larger hematoma volume [88]. Although the current guidelines for pre-hospital ICH management recommend rapid transport of patients with presumed stroke to the closest hospital, more recent approaches include initiation of BP-lowering by the emergency medical services [146,147]. However, some unsolved questions regarding the pre-hospital level still remain, such as at which value and timing the BP should be treated and by how much the BP should be lowered.

At ED admission, rapid and aggressive lowering of BP is usually not recommended due to the detrimental effects on cerebral perfusion in the ischemic penumbra areas [148,149]. Although the benefits of long-term lowering of BP for primary and secondary prevention of ischemic stroke are well established [26,62,150], uncertainty persists in terms of the benefits and risks of early antihypertensive treatment at the onset of ischemic stroke [96,151]. International guidelines emphasize the risks associated with very high SBP (i.e., >220 mmHg) but generally do not recommend reducing BP in ischemic stroke, except for in the context of thrombolysis, where an intermediate level (i.e., SBP <185 mmHg) is considered appropriate to decrease the risk of hemorrhagic transformation of an ischemic lesion. Therefore, given that observational studies reported an increased risk of ICH in patients with markedly elevated BP undergoing IV thrombolysis, a preliminary question in BP management is whether such patients should receive thrombolysis or not [66,73].

Regarding timing, the AHA/ASA guidelines [96] for BP management in acute ischemic stroke have provided recommendations on when the lowering of BP should be optimally started. Namely:

1. Class I recommendation and level B evidence for patients who have elevated BP and are otherwise eligible for treatment with IV rtPA should have their SBP < 185 mmHg and DBP < 110 mmHg before thrombolytic therapy is initiated. If medications are given to lower BP, clinicians should be sure that the BP is stabilized at the lower level before beginning rtPA and maintained <180/105 mmHg for at least the first 24 h after IV rtPA;
2. Class I recommendation and level C evidence are given for patients who have elevated BP but are not eligible for thrombolysis to lower BP by 15% during the first 24 h after onset of stroke. The exact level of BP is not known, but consensus exists that medications should be withheld unless SBP > 220 mmHg or DBP > 120 mmHg;
3. Class IIa recommendation and level B evidence for restarting antihypertensive medications after the first 24 h in patients who have pre-existing hypertension and are neurologically stable, unless a specific contraindication to restarting treatment is known.

In patients with ICH, several trials have demonstrated either a small benefit (INTERACT-2 [81] and ENOS [71]) or no benefit (ATACH-2 [86]) with intensive SBP reduction compared with modest or standard BP reduction. Differences may be explained by the variation in intensity of SBP reduction between trials. A treatment threshold of SBP ≥180 mm with the target goal of reduction to 130–150 mmHg within 6 h of symptom onset may be best choice [152].

In the management of BP in the context of recanalization therapy, less data are available during mechanical thrombectomy [153]. Mechanical thrombectomy is the standard of care for patients who present with an acute ischemic stroke within 6 h of symptom onset and up to 24 h in appropriately selected cases. A recent review [154] concluded that permissive hypertension (defined as <220/120 or <180/105 mmHg, as per the AHA/ASA guidelines) may be harmful in the post-operative period following thrombectomy, especially in patients who were successfully recanalized. Moderate BP control (<160/90 mmHg) was found to be a predictor of improved three-month mortality based on multivariable logistic regression analysis in patients who sustained successful reperfusion. A 10 mmHg increase in SBP resulted in a lower odds ratio of having a favorable three-month functional independence, as well as higher rates of three-month mortality. The authors concluded that BP lower rather than permissive hypertension may be related to improved outcomes, especially in cases of successful reperfusion, although current data are derived from observational studies and future randomized, controlled trials are needed [154].

The optimal post-operative management method for these patients remains uncertain, especially with regard to optimal BP control. In particular, given the vulnerability of the re-perfused tissue due to impaired autoregulation and BBB disruption, as well as the risk of intraprocedural BP drops, the Society of Neuroscience in Anesthesiology and Critical Care recommends maintaining an SBP of 140–180 mmHg [155]. Conversely, a recent multicenter, randomized clinical trial on 76 patients with non-cardioembolic ischemic stroke within 24 h from the onset, who were ineligible for revascularization therapy or with progressive stroke, showed that phenylephrine-induced hypertension (up to an SBP of 200 mmHg) was safe and improved the National Health Institute Stroke Scale score by ≥2 points [156].

### 4.3. Limitations

The findings reviewed here should be viewed in light of some limitations and critical aspects. The first regards the present paper type, which is a narrative review. Given that the original intent was to provide an update of the literature on this topic, the strict criteria of a systematic review were not applied, and therefore a formal detailed analysis of the studies was not performed.

Second, there are several methodological sources of variability across the studies, such as the timing of drug administration after the stroke onset. In this context, ongoing trials, such as the RIGHT-2 trial [144], might shed light on some controversial issues in BP management.

Third, as hypertension affects both small and large vessels, most studies have focused on motor status only and have not considered non-motor outcomes (e.g., the psycho-cognitive implications [157]) of the vascular territory involved, as recently suggested by some transcranial Doppler studies [158,159].

Additionally, the distinction between HU and HE is not shared by all authors, given that both are frequent causes of access to ED (although HU events are significantly more common) and that BP levels alone do not reliably predict the presence of acute hypertension-mediated organ damage, which should be suspected according to the presenting signs and symptoms [160]. However, the most recent international guidelines, i.e., the 2018 ESC and European Society of Hypertension Guidelines [29] and the 2017 North American Guidelines [18] agree on the definitions of HE and HU and on the algorithms for their management and treatment. These two conditions also diverge in terms of short- and long-term prognosis [161] and require separate diagnostic and therapeutic approaches [28].

Another caveat is that the usefulness of some drug recommendations (such as those for labetalol) is limited by the fact that they are not available in all countries.

Lastly, the lack of objective measurements of the damaged tissue in the majority of studies is a critical point. Given the extension of the therapeutic window to perform IV thrombolysis in patients >80 years, more research on the pharmacodynamics of antihypertensive drugs is recommended.

## 5. Conclusions

**Figure 2:**
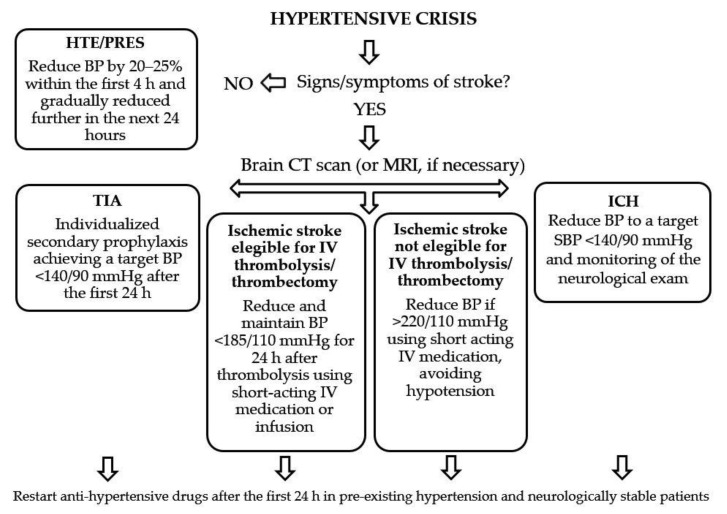
Proposed schematic algorithm to guide the management of blood pressure in patients with TIA, ischemic stroke, and ICH presenting at the emergency department. Legend (in alphabetic order): BP: blood pressure; CT: computed tomography; h: hours; HTE: hypertensive encephalopathy; ICH: intracerebral hemorrhage; IV: intravenous; MRI: magnetic resonance imaging; PRES: posterior reversible leukoencephalopathy syndrome; TIA: transient ischemic attack.

From the studies reviewed here, it clearly emerges that the literature on high BP management during ischemic or hemorrhagic stroke is still controversial. Even systematic reviews and guidelines seem to reach conflicting conclusions, especially regarding BP management in ICH patients. The evidence from recent studies is often contradictory as well, thus making it difficult to provide firm recommendations. To summarize, however, most of the clinical trials on acute ischemic stroke have generally shown a neutral effect of BP reduction on clinical outcomes. Therefore, aggressive BP reduction in patients presenting with acute ischemic stroke is currently not recommended. Although in patients treated with IV rtPA the guidelines recommend a BP < 180/105 mmHg, currently the optimal BP management approach after reperfusion therapy remains unclear. In acute ICH, the evidence from randomized clinical trials supports BP lowering targeting an SBP value of 140 mmHg, which is also recommended in the guidelines [162]. In conclusion, we propose a schematic algorithm (Figure 2) to guide BP management in different clinical settings, i.e., for TIA, ischemic stroke (eligible or not eligible for IV thrombolysis or thrombectomy), and ICH.

## Figures and Tables

**Table 1:** Relevant studies on hypertensive crisis and ischemic stroke.

Study	Design	Patients *n*	Aim/Outcomes	Methods	Main Findings
Barer et al., 1988 [59]	Prospective randomized trial (BEST)	302	To test the protective effect of propranolol on cerebral function in patients with acute stroke.	Patients with clinically diagnosed hemispheric stroke within the previous 48 h were randomly assigned to atenolol, propranolol, or placebo for 3 weeks.	More mortality among patients allocated to β-blockers. The outcome in those taking beta-blockers at the time of stroke was considerably better, suggesting that prior treatment might be protective.
Brott et al., 1998 [60]	Prospective Randomized trial	624	To examine the frequency, course, and treatment of hypertension in the NINDS recombinant tissue plasminogen activator stroke trial.	BP was measured at the time of admission, at randomization, and then 36 times during the first 24 h after randomization to correlate antihypertensive therapy (not randomized) with clinical outcomes.	Treated patients who were hypertensive after randomization and received antihypertensive therapy were less likely to have a favorable outcome at three months than those who were hypertensive and did not receive antihypertensive therapy.
Ahmed et al., 2000 [61]	Prospective INWEST study analysis	250	To correlate nimodipine-induced reduction in BP and an unfavorable outcome in acute stroke with and without adjustment for prognostic variables; to investigate outcome in subgroups with increasing levels of BP reduction.	Patients with ischemic stroke (within 24 h) received placebo, 1 mg/h (low-dose), or 2 mg/h (high-dose) of nimodipine.	BP, but not SBP, reduction was associated with neurological worsening after the intravenous administration of high-dose nimodipine after acute stroke. The results related to low-dose nimodipine were not conclusive.
Arima et al., 2001 [62]	Prospective randomized trial (PROGRESS)	6105	To determine the effects of a flexible BP-lowering regimen with an angiotensin-converting enzyme inhibitor (perindopril) and a diuretic (indapamide) on the risk of stroke and other major vascular events among individuals with a history of stroke or transient ischemic attack.	Patients were randomly assigned to active treatment (perindopril, with the addition of indapamide) or placebo.	The BP-lowering regimen reduced the risk of stroke in both hypertensive and non-hypertensive individuals with a history of stroke or transient ischemic attack. Combination therapy produced larger BP reductions and larger risk reductions than perindopril alone.
Rordorf et al., 2001 [63]	Prospective	13	To assess whether induced hypertension with acute stroke would safely identify a subgroup of patients with a BP-dependent neurologic deficit.	Patients underwent induced hypertension for 30 min using accelerating doses of intravenous phenylephrine within 12 h of stroke onset.	The use of induced hypertension in the context of careful clinical setting was likely to be safe and associated with low morbidity and mortality.
Hillis et al., 2003 [64]	Prospective	15	To evaluate the effects of pharmacologically induced BP elevation on function and perfusion in acute ischemic stroke.	Patients with large diffusion–perfusion mismatch were randomly assigned to BP elevation or conventional management.	Positive effect of induced BP elevation on neurological function, associated with a reduction in hypoperfused tissue on perfusion weighted imaging scans.
Castillo et al., 2004 [65]	Observational study	304	To explore the association of SBP and DBP during acute stroke with early neurological deterioration, infarct volume, neurological outcome, and mortality at three months.	SBP and DBP values on admission and on the first day were the average values of all readings obtained in the emergency department and during a 24 h period after patient allocation in the stroke unit.	High and low SBP and DBP, as well as a relevant drop in BP, were associated with poor prognosis in patients with ischemic stroke. The effects disappeared after adjustment for the use of antihypertensive drugs and BP drop >20 mm Hg within the first day.
Ahmed et al., 2009 [66]	Retrospective analysis of the SITS-ISTR	11,080	To examine the relationship between BP and antihypertensive therapy with outcomes in patients with and without a history of hypertension treated with intravenous thrombolysis using the “Safe Implementation of Thrombolysis in the Stroke—International Stroke Thrombolysis Register” (SITS-ISTR).	Patients were categorized in 4 groups according to a history of hypertension and antihypertensive therapy within 7 days after thrombolysis. BP values were recorded at baseline, 2 h, and 24 h after thrombolysis.	There was a strong association of high SBP after thrombolysis with poor outcome. Antihypertensive therapy up to 7 days in patients with a history of hypertension was associated with worse outcome, whereas initiation of antihypertensive therapy in newly recognized moderate hypertension was associated with a favorable outcome.
Potter et al., 2009 [67]	Prospective randomized trial (CHHIPS)	179	To assess the feasibility, safety, and effects of two regimens for lowering BP in patients who had a ischemic or hemorrhagic stroke.	Patients with cerebral infarction or hemorrhage and hypertension (SBP > 160 mmHg) were randomly assigned to oral labetalol, lisinopril, or placebo if they were non-dysphagic; or to intravenous labetalol, sublingual lisinopril, or placebo if they had dysphagia within 36 h of symptom onset.	Labetalol and lisinopril were effective antihypertensive drugs in acute stroke and did not increase serious adverse events. Early lowering of BP with lisinopril and labetalol after an acute stroke was a promising approach to reduce mortality and future disability.
Robinson et al., 2010 [68]	Prospective randomized trial (COSSACS)	763	To assess the efficacy and safety of continuing or stopping pre-existing antihypertensive drugs in patients who recently had a stroke.	Patients taking antihypertensive drugs enrolled within 48 h of stroke and the last dose of antihypertensive drug and randomly assigned to either continuing or stopping pre-existing antihypertensive drugs for 2 weeks.	Continuation of antihypertensive drugs did not reduce 2-week death or dependency, cardiovascular event rate, or mortality at 6 months. Lower BP levels in those who continued treatment after an acute mild stroke were not associated with more adverse events.
Sandset et al., 2011 [69]	Prospective randomized trial (SCAST)	2029	To examine whether a careful BP-lowering treatment with the angiotensin-receptor blocker candesartan was beneficial in patients with acute stroke and increased BP.	Patients with acute stroke (ischemic or hemorrhagic) and SBP ≥140 mmHg included within 30 h from symptom onset and randomly allocated to candesartan or placebo for 7 days, with increasing dosages.	There was no indication that careful BP-lowering treatment with the angiotensin-receptor blocker candesartan was beneficial in patients with acute stroke and raised BP.
He et al., 2014 [70]	Prospective randomized trial (CATIS)	4071	To evaluate whether an immediate BP reduction in patients with acute ischemic stroke would reduce death or major disability at 14 days or at hospital discharge.	Patients with non-thrombolyzed ischemic stroke within 48 h of onset and elevated SBP were randomly assigned to receive antihypertensive treatment or to discontinue all antihypertensive medications (control) during hospitalization.	Among patients with acute ischemic stroke, BP reduction with antihypertensive medications did not reduce the likelihood of death or major disability at 14 days, or hospital discharge compared with the absence of antihypertensive medication.
Bath et al., 2015 [71]	Prospective randomized trial (ENOS)	4011	To assess the safety and efficacy of glyceryl trinitrate within 48 h in patients with acute ischemic or hemorrhagic stroke and high BP. To assess outcomes for a subset of patients who continued or stopped taking antihypertensive drugs for 1 week after their stroke.	Patients hospitalized with an acute ischemic or hemorrhagic stroke and increased SBP (140–220 mm Hg) were randomly assigned to 7 days of transdermal glyceryl trinitrate (5 mg per day) within 48 h of stroke onset or to no treatment.	In patients with acute stroke and high BP, transdermal glyceryl trinitrate lowered B and had acceptable safety, although it did not improve functional outcome. There was no evidence to support continuing pre-stroke antihypertensive drugs in patients in the first few days after the stroke.
Jusufovic et al., 2015 [72]	Prospective subgroup analysis of SCAST	993	To assess whether the effects of BP lowering with the angiotensin receptor blocker candesartan in the acute phase of stroke were harmful in the subgroup of patients with carotid artery stenosis.	Patients with carotid artery stenosis presenting within 30 h from acute ischemic or hemorrhagic stroke and with SBP ≥140 mmHg were treated with candesartan or placebo for 7 days.	No clear evidence that candesartan was qualitatively different in patients with carotid stenosis, but patients with severe stenosis were at particularly high risk of stroke progression and poor functional outcome.
Wu et al., 2016 [73]	Prospective observational (TIMS-China Registry)	1128	To identify the association between BP and clinical outcomes in acute ischemic stroke patients treated with a thrombolytic medication (recombinant tissue plasminogen activator).	SBP and DBP at baseline, 2 h, and 24 h after treatment and changes from baseline were analyzed in patients hospitalized within 4.5 h from acute ischemic stroke for intravenous thrombolysis.	Lower BP within the first 24 h was associated with a more favorable outcome and less frequent spontaneous ICH in patients with acute ischemic stroke undergoing thrombolytic medication.
Allison et al., 2019 [74]	Retrospective	210	To determine whether clevidipine achieved faster BP control compared to nicardipine in patients with acute ischemic stroke or ICH.	Patients receiving clevidipine or continuous infusion of nicardipine for acute BP management.	No difference in the mean time from infusion initiation to SBP goal between the agents or secondary outcomes (door-to-needle time, length of stay, mortality)
Nasi et al., 2019 [75]	Prospective (MAPAS)	218	To determine the efficacy of the early manipulation of SBP in non-thrombolyzed patients.	Patients randomized within 12 h from acute ischemic stroke to maintain SBP during 24 h within 3 ranges (group 1: 140–160 mmHg; group 2: 161–180 mmHg; group 3: 181–200 mmHg) using vasoactive drugs and fluids.	The modified Rankin Scale at 90 days did not differ among the groups. None had neurological deterioration due to BP reduction in 24 h. ICH occurred more frequently in higher SBP (181–200 mmHg). More chance of good outcome in Group 2.

BEST: Beta Blockade in Acute Stroke; BP: blood pressure; CATIS: China Antihypertensive Trial in Acute Ischemic Stroke; CHHIPS: Controlling Hypertension and Hypotension Immediately Post-Stroke; COSSACS: Continue or Stop Post-Stroke Antihypertensives Collaborative Study; DBP: diastolic blood pressure; ENOS: Efficacy of Nitric Oxide in Stroke; ICH: intracranial hemorrhage; INWEST: Intravenous Nimodipine West European Stroke Trial; MAPAS: Early Manipulation of Arterial Blood Pressure in Acute ischemic Stroke; NINDS: National Institute of Neurological Disorders and Stroke; PROGRESS: perindopril protection against recurrent stroke study; SBP: systolic blood pressure; SCAST: Scandinavian Candesartan Acute Stroke Trial; SITS-ISTR: Safe Implementation of Thrombolysis in the Stroke-International Stroke Thrombolysis Register; TIMS-China: Thrombolysis Implementation and Monitor of Acute Ischemic Stroke in China.

**Table 2:** Relevant studies on hypertensive crisis and intracerebral hemorrhage.

Study	Design	Patients *n*	Aim/Location	Main Findings
Anderson et al., 2008 [76]	Prospective randomized trial (INTERACT)	404	Lobar (9.0%), basal ganglia (82.5), brainstem (4.5%), cerebellum (4%), intraventricular extension (23.5%).	The relative risk of hematoma growth was lower with an intensive BP-lowering treatment. Clinical outcomes were not different with intensive blood-pressure-lowering treatment.
Koch et al., 2008 [77]	Prospective	42	Feasibility and safety of reducing BP to lower than presently recommended levels in acute ICH.	Aggressive lowering of BP did not affect hematoma, edema expansion, neurological deterioration, or outcome.
Suri et al., 2008 [78]	Retrospective	122	Drop in systolic BP and mean arterial pressures over 24 h were divided into quartiles to determine the risk of neurological deterioration among quartiles.	The reduction of BP in patients with acute ICH is safe. An aggressive decrease in BP might reduce the risk of neurological deterioration in the first 24 h of admission.
Anderson et al., 2010 [79]	Prospective randomized trial (INTERACT)	404	To determine the effects of intensive BP reduction on hematoma and perihematomal edema over 72 h.	The early intensive BP-lowering treatment attenuated hematoma growth over 72 h. There was no appreciable effect on perihematomal edema.
Qureshi et al., 2010 [80]	Prospective (ATACH)	774	Lobar hemorrhages.	Reduced mortality rate with systolic BP-lowering treatment.
Anderson et al., 2013 [81]	Prospective randomized trial (INTERACT-2)	2839	Deep location of hematoma (83.5%), intraventricular extension of hemorrhage (28.3%).	Death or severe disability was not reduced with intensive BP-lowering treatment. Lower modified Rankin scale with intensive BP-lowering treatment.
Butcher et al., 2013 [82]	Prospective randomized trial (ICH ADAPT)	75	Basal ganglia (74.5%), lobar (22.5%), brainstem (3.0%), intraventricular extension (38.5%).	Acute BP lowering did not worsen cerebral ischemia.
Sakamoto et al., 2013 [83]	Prospective, observational (SAMURAI)	211	Putamen (57%), thalamus (36%), lobar (7%).	Aggressive BP lowering may ameliorate clinical outcomes.
Rodriguez-Luna et al., 2013 [84]	Prospective	117	Supratentorial, intraventricular extension of hemorrhage (47%).	Systolic BP and variability predict hematoma growth and early neurological deterioration.
Krishnan et al., 2016 [85]	Prospective randomized trial (ENOS subanalysis)	246	To continue or stop antihypertensive treatment during the acute phase of ICH.	Among patients with acute ICH, immediate continuation of antihypertensive drugs during the first week did not reduce death or major disability in comparison to stopping treatment temporarily.
Qureshi et al., 2016 [86]	Prospective randomized trial (ATACH-2)	1000	Thalamus (37.8%), basal ganglia (51.2%), cerebral lobe (11.0%), cerebellum (0.1%)	A target BP values from 110 to 139 mmHg did not result in a lower rate of death or disability than a standard reduction.
Bozzano et al., 2017 [87]	Randomized, controlled, multicenter trial	1000	To determine the efficacy and safety of early and rapid BP lowering in patients with spontaneous ICH.	No clinical benefits from intensive and rapid lowering of BP.
Hatcher et al., 2017 [88]	Retrospective observational	243	Intraventricular extension of hemorrhage (64%), infratentorial bleeding (20%).	Elevated pre-hospital systolic BP (≥140 mmHg) was associated with larger hematoma volumes.
Rodriguez-Luna et al., 2018 [89]	Retrospective	219	Lobar hemorrhages (40.6%). Intraventricular extension (48.9%), subarachnoid extension (35.2%).	Pre-hospital BP was correlated with hematoma volume at admission.
Zhu et al., 2020 [90]	Single-center retrospective study	166	To compare early versus late initiation of oral antihypertensives on intensive care unit length of stay and cost of hospitalization in patients with ICH.	Early initiation of oral antihypertensives is safe and may have significant clinical and socio-economic impacts on patients with hypertensive ICH.

ATACH: Antihypertensive Treatment of Acute Cerebral Hemorrhage; BP: blood pressure; ICH: intracerebral hemorrhage; ICH ADAPT: Intracerebral Hemorrhage Acutely Decreasing Arterial Pressure Trial; ENOS: Efficacy of Nitric Oxide in Stroke; INTERACT: Intensive Blood Pressure Reduction in Acute Cerebral Haemorrhage Trial; INTERACT-2: Second Intensive Blood Pressure Reduction in Acute Cerebral Haemorrhage Trial; SAMURAI: Stroke Acute Management with Urgent Risk-Factor Assessment and Improvement- Intracerebral Hemorrhage Study.
